# Natural Immunity to HIV is associated with Low BLyS/BAFF levels and low frequencies of innate marginal zone like CD1c^+^ B-cells in the genital tract

**DOI:** 10.1371/journal.ppat.1007840

**Published:** 2019-06-07

**Authors:** Lyvia Fourcade, Catherine Sabourin-Poirier, Victoire Perraud, Marie-Claude Faucher, Josiane Chagnon-Choquet, Annie-Claude Labbé, Michel Alary, Fernand Guédou, Johanne Poudrier, Michel Roger

**Affiliations:** 1 Laboratoire d’Immunogénétique, Centre de Recherche du Centre Hospitalier de l’Université de Montréal (CRCHUM), Montréal, Canada; 2 Département de Microbiologie, Infectiologie et Immunologie de l‘Université de Montréal, Montréal, Canada; 3 Département de Microbiologie Médicale et Infectiologie, Hôpital Maisonneuve-Rosemont, Montréal, Canada; 4 Centre de recherche du CHU de Québec–Université Laval, Québec, Canada; 5 Département de Médecine Sociale et Préventive, Université Laval, Québec, Canada; 6 Institut National de Santé Publique du Québec, Québec, Canada; 7 Dispensaire IST, Cotonou, Bénin; Vaccine Research Center, UNITED STATES

## Abstract

BLyS/BAFF is recognized for its role in B-cell ontogenesis, as well as cell fate decision towards the first-line/innate marginal zone (MZ) B-cell pool. Excess BLyS/BAFF is associated with hyperglobulinemia and increased frequencies of activated precursor-like MZ B-cells. Herein, we show that HIV highly-exposed seronegative (HESN) commercial sex workers (CSWs) had lower soluble BLyS/BAFF levels and relative frequencies of BLyS/BAFF expressing cells in their genital mucosa when compared to those from HIV-infected CSWs and HIV-uninfected non-CSWs. Furthermore, we identified genital innate and/or marginal zone (MZ)-like CD1c^+^ B-cells that naturally bind to fully glycosylated gp120, which frequencies were lower in HESNs when compared to HIV-infected CSWs and HIV-uninfected non-CSWs. Although genital levels of total IgA were similar between groups, HESNs had lower levels of total IgG1 and IgG3. Interestingly, HIV-gp41 reactive IgG1 were found in some HESNs. Low genital levels of BLyS/BAFF observed in HESNs may allow for controlled first-line responses, contributing to natural immunity to HIV.

## Author summary

Worldwide, most human immunodeficiency virus (HIV) infections affect women through heterosexual intercourse. We and others have identified African female commercial sex workers (CSWs), who remain seronegative despite high exposition to HIV (HESNs). Innate marginal zone (MZ) B-cells recirculate in humans and have been found in front-line areas such as the sub-epithelial lamina propria of mucosal associated lymphoid tissues. MZ B-cells can bind to fully glycosylated gp120 and produce specific IgG and IgA, and have a propensity for B regulatory potential, which could help both the fight against HIV and maintenance of low inflammatory conditions reported for HESNs. Here we identify genital MZ-like B-cells, which frequencies are lower in the genital tract of HESNs when compared to HIV-infected CSWs and HIV-uninfected non-CSW women. Furthermore, this coincides with significantly lower genital levels of B lymphocyte stimulator (BLyS/BAFF), known to shape the MZ pool and which overexpression leads to MZ deregulation in HIV-infected progressors. HESN individuals provide an exceptional opportunity to determine important clues for the development of protective devices. Here we show that contained BLyS/BAFF levels are concomitant with natural immunity against HIV, and may prevent dysregulated first-line responses. MZ-like B-cells could be harnessed in preventive strategies viewed at soliciting quick first-line to be adjunct to matured long term protection.

## Introduction

Worldwide, most HIV infections are acquired through heterosexual intercourse, and in sub-Saharan Africa, 60% of new HIV infections affect women [1]. Observations made in the context of natural immunity to HIV may help identify important clues for the development of protective devices. As such, we established a cohort of female commercial sex workers (CSWs), in Cotonou (Benin), in which we have identified HIV highly-exposed seronegative (HESN) individuals, who remain uninfected after more than 4 years of active prostitution. Beninese HESN CSWs have significantly lower genital levels of pro-inflammatory cytokines and chemokines when compared to both HIV-infected CSWs and HIV-uninfected non-CSWs [2, 3]. Previous studies from Kenyan female CSWs demonstrated that HESNs have a low activation T-cell profile in both the blood and vaginal mucosa, which corresponds with a greater ability to proliferate in response to HIV-p24 peptides when compared to HIV-infected CSWs [4–7]. Furthermore, we and others have demonstrated elevated frequencies of T-regulatory lymphocytes in the blood [8] and genital tract [9] of HESN CSWs, the latter which were concomitant with increased frequencies of dendritic cells (DC) bearing a tolerogenic profile. Altogether, these findings suggest that the capacity to regulate the activation/inflammatory profile is associated with protection against HIV infection.

Consistent with their low-inflammatory profile, we recently reported that Beninese HESNs have lower levels of B Lymphocyte Stimulator (BLyS/BAFF) in their blood when compared to HIV-uninfected non-CSWs [10]. BLyS/BAFF is highly recognized for its role in B-cell ontogenesis, as well as cell fate decision towards the first-line/innate marginal zone (MZ) B-cell pool [11, 12]. As such, HESNs have reduced frequencies of mature MZ B-cells in their blood when compared to HIV-uninfected non-CSWs [10]. In contrast, HIV-infected CSWs have higher levels of BLyS/BAFF, hyperglobulinemia and increased frequencies of activated precursor-like MZ B-cells in their blood when compared to those in HESNs [10]. These findings suggest that control of BLyS/BAFF and innate B-cell status could play a role in natural immunity against HIV infection.

Based on these observations, we have now assessed BLyS/BAFF expression levels and innate B-cell status in the genital tract of these women, which is a main portal of entry for HIV. We have been using the “lipid presenting” MHC class I-like molecule CD1c [13], which is a marker shared by “innate-like” populations, to help track MZ-like B-cells [11, 12]. In the present study, we show that as for blood, HESNs have lower levels of BLyS/BAFF and MZ-like CD1c^+^ B-cells in their genital tract when compared to HIV-infected CSWs and HIV-uninfected non-CSWs.

## Materials and methods

### Study groups

Female CSWs were recruited through a dedicated sex worker clinic in Cotonou, Benin. HIV-uninfected non-CSW control women at low risk for exposure were enrolled from a general health clinic in Cotonou. Women were invited to participate in the study as they attended clinics. Women were excluded from the study if, they were less than 18 years old, menstruating or pregnant. At enrolment, participants were asked to answer a questionnaire about demographic information, sexual behavior, duration of sex work, number of sex partners, condom use, vaginal douching practices, and reproductive history. Each participant underwent a genital examination by a physician. Vaginal specimens were obtained for diagnosis of candidiasis, trichomoniasis and bacterial vaginosis by microscopic examination and herpes simplex virus (HSV) infection by PCR. Endocervical swabs were obtained to test for *Neisseria gonorrhoeae* and *Chlamydia trachomatis* infection using BD ProbeTec ET system (Strand Displacement Assay, Becton Dickinson, Heidelberg, Germany). Peripheral blood was taken for HIV, syphilis, HSV and progesterone testing by immunoassays. HIV-1 positivity was defined by the presence of HIV specific IgG tested with Vironostika HIV Uni-Form II Ag/Ab (Organon Teknika, Boxtel, The Netherlands). Non-reactive samples were considered HIV seronegative, whereas reactive samples were tested with Genie II HIV-1/HIV-2 (Bio-Rad, Hercules, CA). Genie II dually reactive samples (to HIV-1 and HIV-2) and discordant samples (Vironostika reactive/Genie II non-reactive) were further tested by INNO-LIA HIV I/II Score (Innogenetics NV, Technologiepark 6, Gent, Belgium). HSV infection and shedding was determined by testing for the presence of HSV in the CVLs of the women by PCR assay. For the present study, we selected samples from 10 HIV-uninfected and 11 treatment-naïve HIV-infected CSWs, and 10 HIV-uninfected non-CSW control subjects from the general population. None of these women were injecting drug users. The three study groups were all in the follicular phase of their menstrual cycle, as determined by blood progesterone levels, not taking oral contraception or injectable contraception such as DMPA or implanted ring, had no HSV, *N gonorrhoeae*, *C trachomatis* infection, bacterial vaginosis, trichomoniasis or candidiasis.

### Ethics statement

Written informed consent was obtained from all subjects who participated in the study. The methods reported in this paper were performed in accordance with the relevant guidelines and regulations and all experimental protocols were approved by the Comité National Provisoire d’Éthique de la Recherche en Santé in Cotonou and the Centre Hospitalier de l’Université de Montréal (CHUM) Research Ethics Committees.

### CVL sample collection and preparation

Mucus was removed initially prior to performing the Cervico-vaginal lavage (CVL). CVL samples were obtained from all study participants by a physician, using a 10-ml syringe filled with sterile 1x phosphate-buffered solution (PBS) and aimed directly into the cervical os. CVL fluids were then collected, transferred immediately into 20 ml of RPMI-1640, kept on ice, and processed within 1 hour. CVL samples were centrifuged at 1500 rpm for 10 min and supernatants were concentrated on a 3 KDa Amicon membrane and stored at -80°C. The CVL cellular fractions were cryopreserved in liquid nitrogen.

### Determination of soluble BLyS/BAFF concentrations in CVL supernatants

BLyS/BAFF levels were determined by using a commercial ELISA kit, R&D systems (Minneapolis, USA).

### Flow-cytometry characterization of BLyS/BAFF surface expression on CVL epithelial cells, T-cells, myeloid DCs, CD14^+^CD11c^+^ “monocytic” or monocyte-derived cells and granulocytes

CVL cells were thawed and washed with RPMI 1640 followed by 1X PBS. Briefly, a maximum of 2×10^5^ cells per well were used for staining. Live/dead exclusion was performed using Aqua-LIVE/DEAD Fixable Stain (Invitrogen Life technologies, Eugene, OR, USA). Non-specific binding sites were blocked using fluorescence-activated cell sorting (FACS) buffer (1x PBS, 2% heat inactivated (hi)-FBS, and 0.1% sodium azide) supplemented with 20% hi-FBS and 10 ug mouse IgG (Sigma-Aldrich, St-Louis, MO, USA). CVL cells were stained using the following conjugated mouse anti-human monoclonal antibodies: BUV395 anti-CD45 and BV786 anti-CD14 (BD-Biosciences, San Jose, CA, USA), PeCy5.5 anti-CD11c, PeCy7 anti-CD66b, AlexaFluor 700 anti-CD3 and PE anti-BLyS (ebiosciences, San Jose, CA, USA), APC CDK PAN cytokeratin for epithelial cells (Cedarlane, Burlington, ON, CA). CVL cells were fixed with 1.25% paraformaldehyde and kept at 4°C for a minimum of 12 hours before flow-cytometry analysis. Briefly, live epithelial cells and leucocytes were analyzed after FSC/SSC gating to remove debris, and removal of doublets, for HIV-infected CSWs: epithelial cells constituted 18% and leukocytes 48% of recovered live cells, which summed to a mean of 17 336 ±6743 total events. For HESNs: epithelial cells constituted 9,3% and leukocytes 37% of recovered live cells, which summed to a mean of 14 867±8938 total events. For HIV-uninfected non-CSWs: epithelial cells constituted 8% and leucocytes 26% of recovered live cells, which summed to a mean of 5650±992 total events. Acquisition was with an LSRFortessa (BD-Biosciences, San Jose, CA, USA) and analyzed with FlowJo7.6.3 software (TreeStar, Ashland, OR, USA). Flow-cytometry data analysis quadrants were set based on the expression values obtained with fluorescence minus one (FMO) and isotype controls.

### Flow-cytometry characterization of total B-cells, plasmablasts and plasma cells in the CVL cellular fraction

Cell processing, staining and analysis were performed as mentioned above. The following conjugated mouse anti-human monoclonal antibodies were used: BUV395 anti-CD45, BUV737 anti-CD138, BV605 anti-CD19, APC/H7 anti-IgG and APC anti-CD1a (BD-Biosciences, San Jose, CA, USA), PerCP efluor710 anti-CD1c and AlexaFluor 700 anti-CD3 (ebiosciences, San Jose, CA, USA). Cells were pre-incubated or not with mannose (5 ug) for 40 minutes on ice, followed by incubation with or without fully glycosylated biotinylated gp120 IIIB (ImmunoDX Inc) at 5 ug/ml for 40 minutes on ice prior to adding the staining cocktail and streptavidin-PE (BD-Biosciences, San Jose, CA, USA).

### Determination of immunoglobulin isotypes concentrations in CVL supernatants

Levels of total immunoglobulin (Ig) isotypes IgG1, IgG2, IgG3, IgG4, IgM and IgA were measured in CVL supernatants using the multiplex bead assay Milliplex Map Kit with human immunoglobulin isotyping Magnetic Bead panel by EMD Millipore (Billerica, USA) according to manufacturer’s protocol. Analysis was performed on a Luminex 200 System (Luminex Corporation, Austin, TX, USA). HIV-gp120 and -gp41 Ig reactivity was detected based on the method previously described [14, 15]. Briefly, non-concentrated CVL supernatants were incubated for 18 hours at 4°C rotating with protein G-agarose (ThermoFisher), and eluted with elution buffer (ThermoFisher) to recover IgG. Subsequently, remaining supernatants were incubated for 18 hours at 4°C rotating with peptide M-agarose (Invivogen) and eluted to recover IgA. IgG and IgA recovery in eluates and presence within remaining supernatants were verified by performing human total IgG and IgA ELISAs (ThermoFisher). The remaining supernatants following IgA recovery were used to detect IgM reactivity. Eluates were neutralized with TRIS 1M pH 7.5 (ThermoFisher) and incubated with gp120 M.CONS-D11 and MN gp41 (NIH AIDS-Reagent program) coated magnetic microspheres (Radix) for 18 hours at 4°C rotating, followed by incubation with either of PE conjugated mouse anti-human IgG1, IgG2, IgG3, IgG4 (Southern Biotech), IgA1, IgA2 and IgM (ebioscience), and detection by Luminex 200 system. The cut–off for positivity was set at mean fluorescence intensity value obtained for 10 HIV-uninfected non-CSWs + 3 SD.

### Statistical analyses

Data from HESNs were compared separately to those of HIV-infected CSWs and HIV-uninfected non-CSWs. The statistical significance of difference between groups was determined by Fisher’s exact test for categorical variables and Unpaired T-test or Mann-Whitney U test analysis for continuous variables. The D’Agostino-Pearson normality test was used to determine whether the values were sampled from a Gaussian distribution. Analyses were performed using GraphPad Prism 5.00 for Windows (GraphPad Software, San Diego, California, USA).

## Results

### Socio-demographic characteristics of the study groups

The socio-demographic characteristics of female CSWs and non-CSWs are shown in Table 1. There were no statistical differences for age between HESNs and the two other groups. All women were practicing vaginal douching. Duration of sex work, average number of clients and condom use were not significantly different between the HESN and HIV-1-infected CSW groups. Thus CSWs and non-CSWs were comparable in terms of socio-demographic characteristics.

**Table 1:** Distribution of demographic and sexual behavior characteristics in HIV-1 uninfected non-CSW, HESN and HIV-1 infected CSW women.

	HIV-1 uninfected non CSWs	HESNs	HIV-1 infected CSWs	*p-value
	N = 10	N = 10	N = 11	
Age, mean (SD), years	38 (11)	36 (9)	41 (9)	NS
Duration of sex work, mean (SD), years	NA	3,6 (1.3)	5,5 (5)	NS
Number of client past week, mean (SD)	NA	18 (15)	13 (10)	NS
Condom always used with client past week	NA	8	5	NS
Vaginal douching	10	10	11	NS

*p-value for comparisons between HESNs and the two other groups were calculated with Mann Whitney U test for age and duration of sex work; Unpaired T-test for the number of clients; Fisher’s exact test for condom use and vaginal douching. CSWs, commercial sex workers; HIV, human immunodeficiency virus; HESN, HIV Highly-Exposed Seronegative; N, number of participants; NA, non-applicable; NS, non-significant; SD, standard deviation.

#### Levels of expression of BLyS/BAFF in CVL supernatants and by genital cell populations of HIV-uninfected non-CSWs, HESNs and HIV-infected CSWs

**Fig 1:**
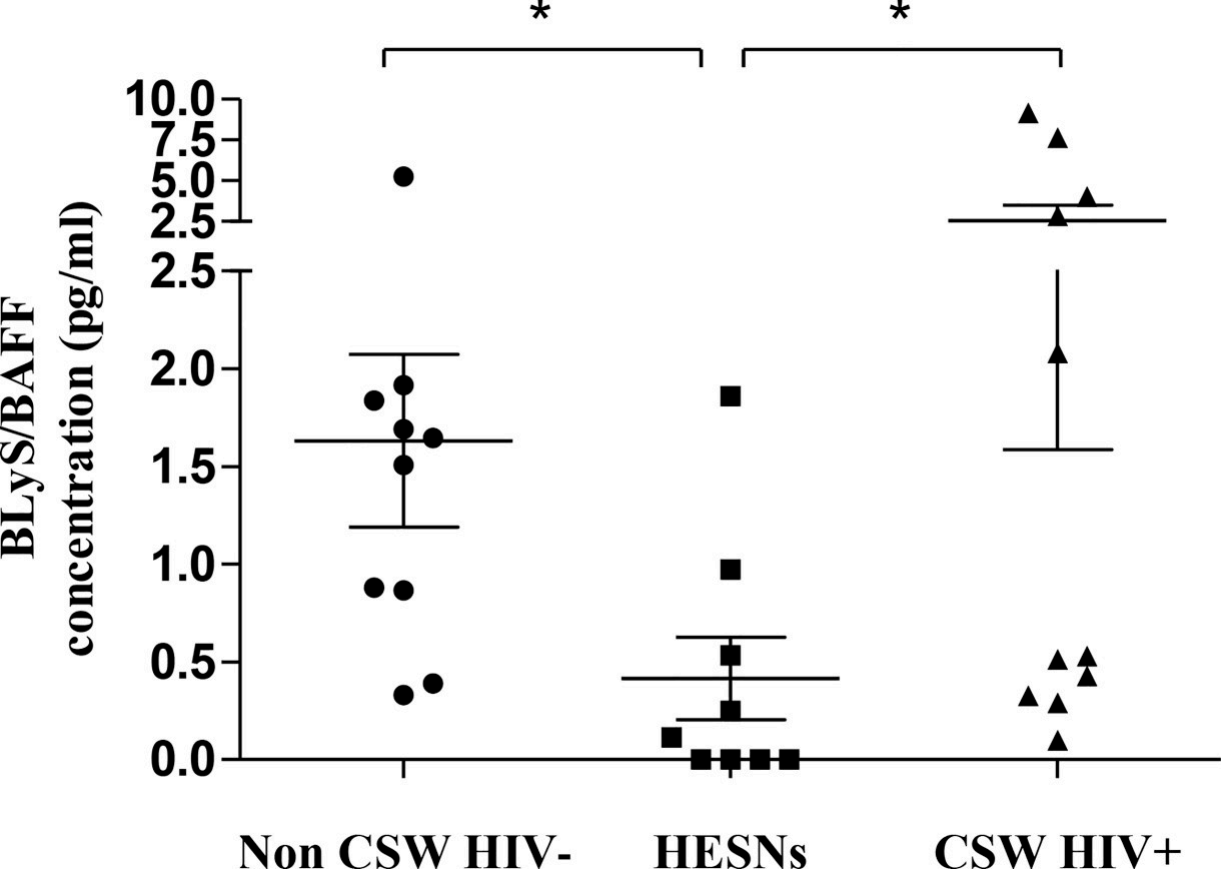
Concentration of BLyS/BAFF in cervicovaginal lavage (CVL) supernatants of HIV-1 uninfected non-CSW, HESN and HIV-1 infected CSW women. Concentrations of BLyS/BAFF (pg/ml) in CVL supernatants were compared with Mann Whitney U test for pair-wise comparisons between HESN and the two other groups. Data are presented as mean ± SD. Significance levels are shown as *(p < 0.05). HIV, human immunodeficiency virus; CSW, commercial sex worker; HESN, HIV Highly-Exposed Seronegative; SD, standard deviation.

**Fig 2:**
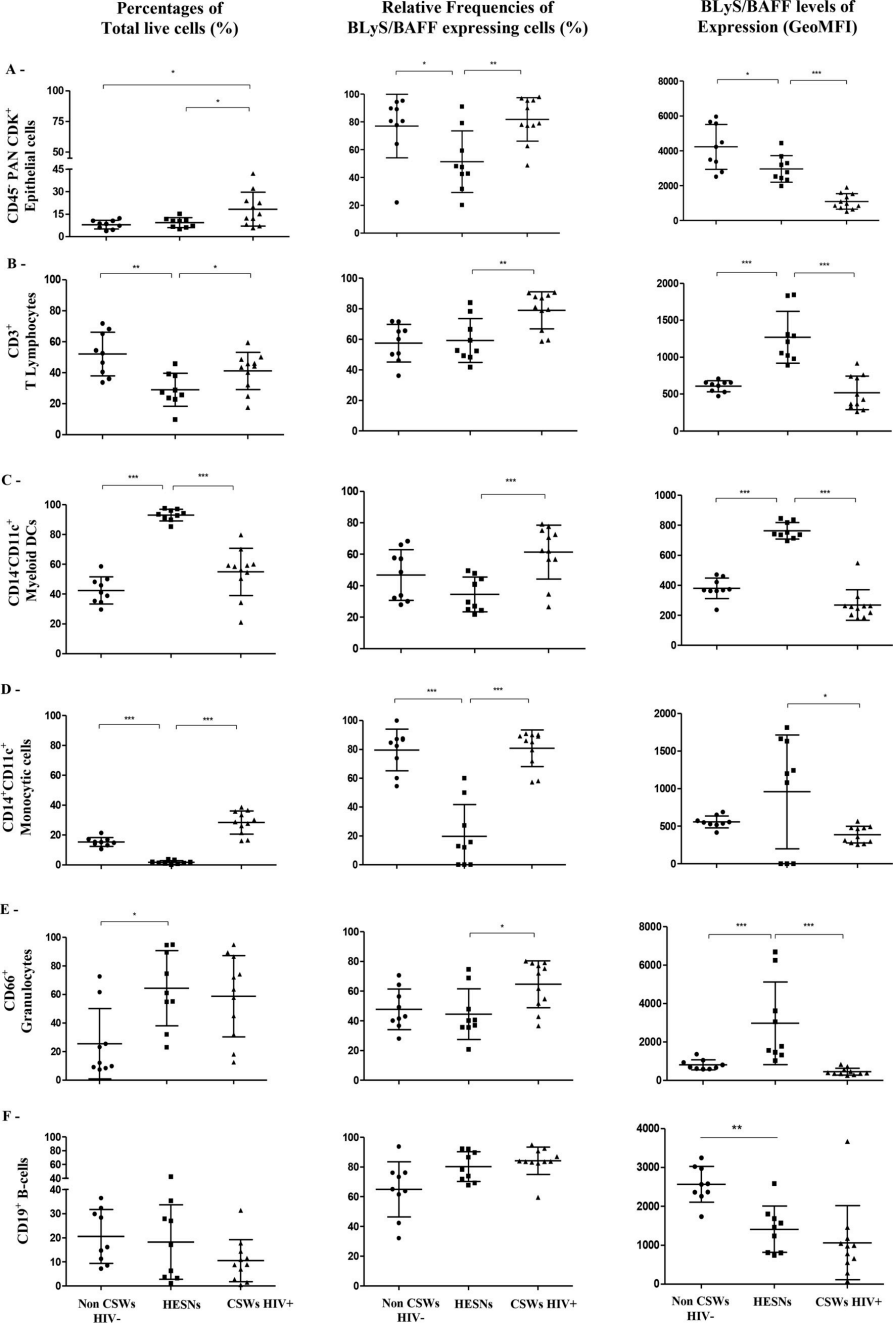
Relative frequencies of BLyS/BAFF expressing cells and surface expression levels in cervicovaginal lavage (CVL) cellular populations of HIV-1 uninfected non-CSW, HESN and HIV-1 infected CSW women. Percentages (%) of total live cells (left panels), relative frequencies of BLyS/BAFF expressing cells (middle panels) and levels of expression (geometric mean fluorescence intensity; GeoMFI) (right panels) were assessed by flow-cytometry. Total cells were gated on live (A) CD45-CDKPAN+ Epithelial cells, and live CD45+CDKPAN- (B) CD3+ T Lymphocytes, (C) CD11c+CD14- myeloid Dendritic cells (DCs), which were negative for CD3, CD19,CD56 and CD66, (D) CD11c+CD14+ “Monocytic” monocyte derived cells, which were negative for CD3, CD19,CD56 and CD66, and (E) CD66+ Granulocytes and (F) B-cells. BLyS/BAFF expressing cells were gated from total respective populations. Data are presented as mean ± SD. Statistical significance of differences in the relative frequencies (%) and levels of expression (GeoMFI) were evaluated with Mann Whitney U test for pair-wise comparisons between HESNs and the two other groups. *p < 0.05, **p < 0.01 and ***p < 0.001. Frequencies (%) of total live cell populations (left panels) are calculated vs total live CVL cells (A) or total live CD45+ CDKPAN- cells (B-F). Relative frequencies (%) of BLyS/BAFF expressing cells (middle panels) are calculated vs frequencies (%) of total respective populations (left panels). HIV, human immunodeficiency virus; CSWs, commercial sex workers; HESN, HIV Highly-Exposed Seronegative. SD, standard deviation.

We have previously shown that BLyS/BAFF levels were lower in the blood of HESNs when compared to the two other groups. As with the blood compartment, we found that BLyS/BAFF levels measured in CVL supernatants of HESNs were significantly lower than those observed in both HIV-uninfected non-CSWs and HIV-infected CSWs (Fig 1). Because determining and comparing frequencies of cells expressing BLyS/BAFF might be influenced by the fluctuations in cell populations between the study groups [10, 16], we have assessed the percentages (Fig 2, left panels) of total: (A) epithelial cells, (B) T-cells, (C) CD14^-^CD11c^+^ myeloid DCs, (D) CD14^+^CD11c^+^ “monocytic” or monocyte-derived cells, (E) granulocytes and (F) B-cells in the CVL cellular fractions of HIV-uninfected non-CSWs, HESNs and HIV-infected CSWs. Gating strategies are found in S1 Fig. We found that the relative percentages of epithelial cells in HESNs were comparable to that of HIV-uninfected non-CSWs and lower than that of HIV-infected CSWs (Fig 2A left panel). Percentages of T-cells were significantly lower in HESNs when compared to HIV-uninfected non-CSWs and to HIV-infected CSWs (Fig 2B left panel). When compared to those in HIV-uninfected non-CSWs, percentages of granulocytes were elevated in HESNs, and similar to those of HIV-infected CSWs (Fig 2 E left panel). Percentages of myeloid DCs were higher in HESNs when compared to those in both HIV-uninfected non-CSWs and HIV-infected CSWs (Fig 2 C left panel). Percentages of CD14^+^CD11c^+^ “monocytic” cells were decreased in HESNs when compared to both HIV-uninfected non-CSWs and HIV-infected CSWs (Fig 2D left panel). The relative percentages of cells expressing BLyS/BAFF within these populations were significantly or tended to be lower in HESNs when compared to HIV-uninfected non-CSWs and HIV-infected CSWs (Fig 2A and 2C–2E middle panels), except for T-cells, which relative percentages of BLyS/BAFF expressing cells were comparable to that of HIV-uninfected non-CSWs and lower than that of HIV-infected CSWs (Fig 2B middle panel). Strikingly, we found higher cell surface expression levels of BLyS/BAFF in HESNs when compared to HIV-uninfected non-CSWs and HIV-infected CSWs (Fig 2B–2E right panels), except for epithelial cells and B-cells, which levels of expression were lower when compared to HIV-uninfected non-CSWs, but higher than HIV-infected CSWs (Fig 2A and 2F right panels). Thus, although the genital cells of HESNs expressed relatively higher levels of BLyS/BAFF than the cells of the other groups of women, HESNs had lower levels of soluble BLyS/BAFF and lower relative frequencies of BLyS/BAFF expressing cells in their genital mucosa.

#### Frequencies of plasmablasts and plasma cells in the genital mucosa of HIV-uninfected non-CSWs, HESNs and HIV-infected CSWs

**Fig 3:**
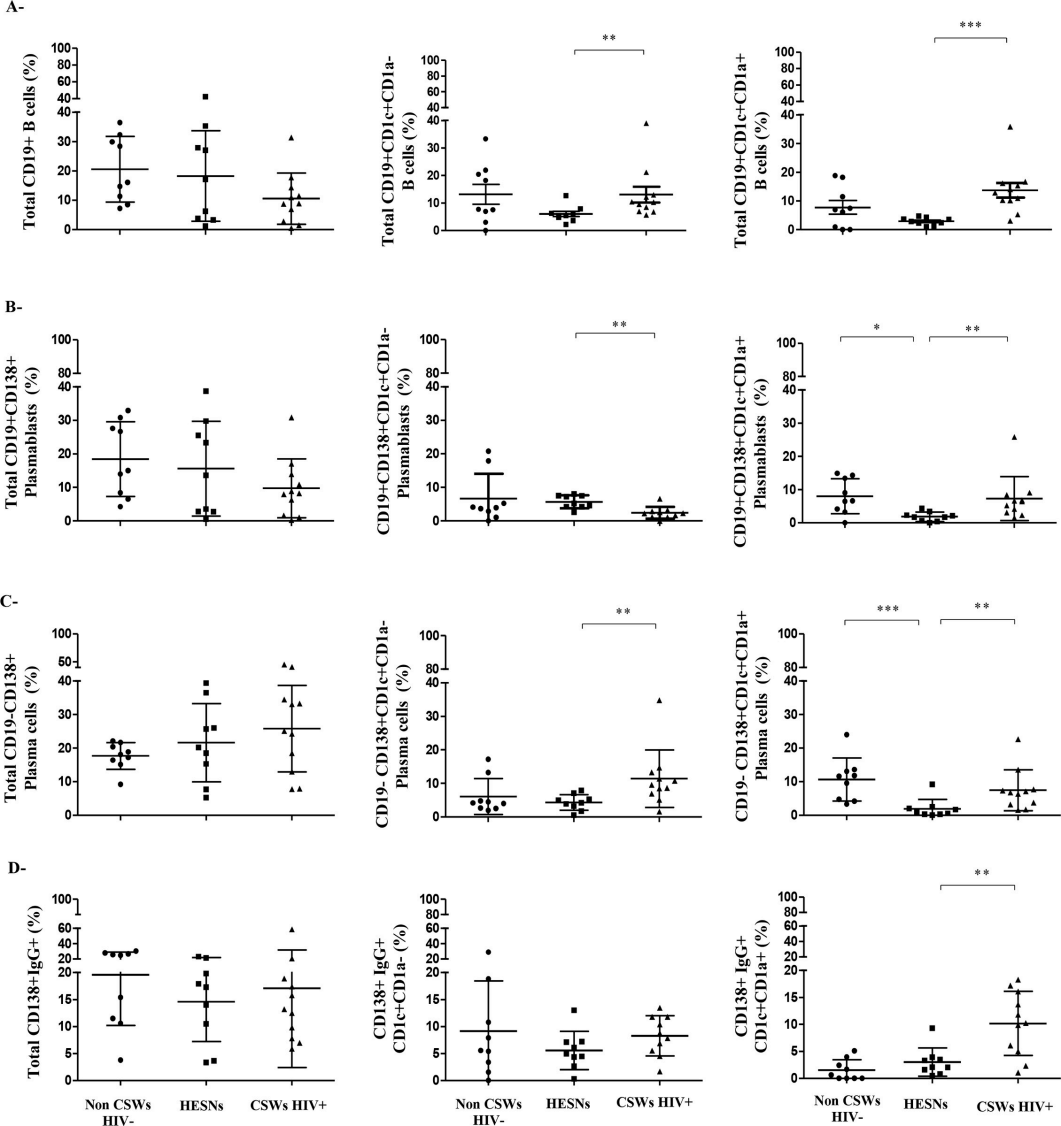
Frequencies of total B-cells, plasmablasts and plasma cells in the cervicovaginal lavage (CVL) cellular fraction of HIV-1 uninfected non-CSW, HESN and HIV-1 infected CSW women. Flow-cytometry analysis of (A) total CD19+ (left panel), CD19+CD1c+CD1a- (middle panel), and CD19+CD1c+CD1a+ (right panel) B-cells, (B) total CD19+CD138+ (left panel), CD19+CD138+CD1c+CD1a- (middle panel) and CD19+CD138+CD1c+CD1a+ (right panel) plasmablasts, (C) total CD19-CD138+ (left panel), CD19-CD138+CD1c+CD1a- (middle panel) and CD19-CD138+CD1c+CD1a+ (right panel) plasma cells, (D) total CD138+IgG+ (left panel), CD138+IgG+CD1c+CD1a- (middle panel) and CD138+IgG+CD1c+CD1a+ (right panel) plasmablasts and plasma cells. Cells were gated on live CD45+ CDKPAN- cells and were negative for CD3, CD56 and CD66. Relative frequencies were calculated vs total live CD45+CDKPAN- cells (A,B,C and D) left panels, vs total CD19+ B-cells (A) middle and right panels, vs total CD138+CD19+plamablasts (B) middle and right panels, vs total CD19-CD138+ plasma cells (C) middle and right panels, and vs CD138+IgG+ plasmablasts and plasma cells (D) middle and right panels. Data are presented as mean ± SD. Statistical significance of differences in the relative frequencies (%) were evaluated with Mann Whitney U test for pair-wise comparisons between HESNs and the two other groups. *p < 0.05, **p < 0.01 and ***p < 0.001. HIV, human immunodeficiency virus; CSWs, commercial sex workers; HESN, HIV Highly-Exposed Seronegative. SD, standard deviation.

Variability in BLyS/BAFF expression levels may impact on B-cell populations. As such, we expected that B-cells such as MZ populations, which development and activation involve signals from BLyS/BAFF [11], be influenced by the differences of BLyS/BAFF expression we observed between HESNs and the two other groups. There were no significant differences when comparing the relative frequencies of total B-cells (CD19^+^), plasmablasts (CD19^+^CD138^+^) and plasma cells (CD19^-^CD138^+^) in CVL cellular fractions between HESNs and the two other groups (Fig 3A, 3B and 3C left panels). Gene expression analyses of blood MZ B-cells showed that CD1a expression allowed to further differentiate “mature” CD1c^+^CD1a^-^ from “precursor-like” CD1c^+^CD1a^+^ MZ B-cell populations (S2A Fig). The relative frequencies of total innate MZ-like CD19^+^CD1c^+^CD1a^-^ and CD19^+^CD1c^+^CD1a^+^ B-cells were lower in HESNs when compared to both HIV-uninfected non-CSWs and HIV-infected CSWs (Fig 3A middle and right panels). The relative percentages of CD1c^+^CD1a^-^ plasmablasts in HESNs were comparable to that of HIV-uninfected non-CSWs and greater than that of HIV-infected CSWs (Fig 3B middle panel), whereas frequencies of CD1c^+^CD1a^-^ plasma cells in HESNs were lower when compared to HIV-infected CSWs (Fig 3C middle panel). Both CD1c^+^CD1a^+^ plasmablasts and plasma cells were significantly lower in HESNs when compared to the two other groups (Fig 3B and 3C right panels). There were no significant differences in IgG-expression by total CD138^+^ or CD1c^+^CD1a^-^ plasmablasts and plasma cells between the three study groups (Fig 3D left and middle panels). However there were less IgG expressing CD1c^+^CD1a^+^ B-cells in HESNs when compared to those of HIV-infected CSWs (Fig 3D right panel). Gating strategies are found in S3 Fig. Thus, consistent with lower levels of soluble BLyS/BAFF, HESNs had reduced frequencies of innate MZ-like CD1c^+^ B-cells in their genital tract when compared to HIV-infected CSWs and HIV-uninfected non-CSWs. Furthermore, there were less IgG expressing CD1c^+^CD1a^+^ B-cells in HESNs when compared to those of HIV-infected CSWs.

**Fig 4:**
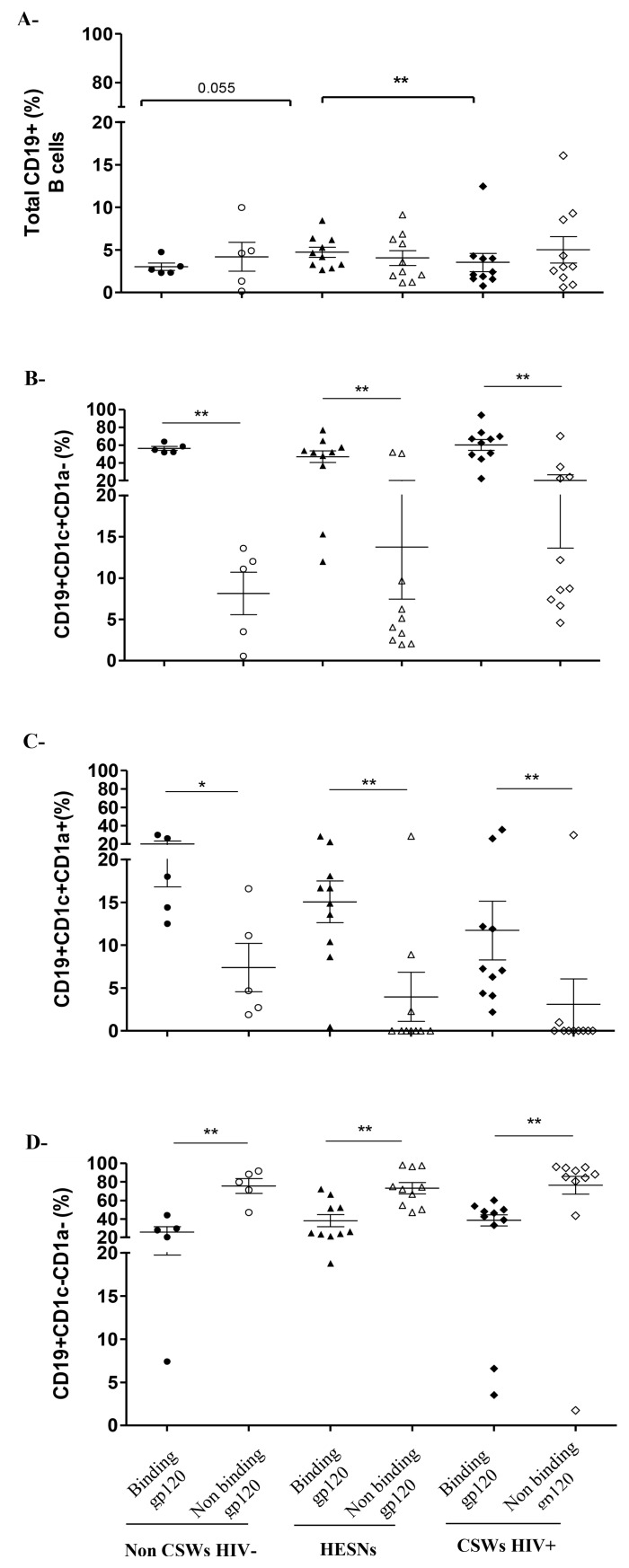
Analysis of gp120 binding by B-cell populations in the cervicovaginal lavage (CVL) cellular fraction of HIV-1 uninfected non-CSW, HESN and HIV-1 infected CSW women. Cells were pre-incubated with fully glycosylated gp120 IIIB and processed for flow-cytometry. Data are represented as gp120 binding (filled) vs non-binding (clear) (A) total CD19+ B-cells, (B) CD19+CD1c+CD1a- B-cells, (C) CD19+CD1c+CD1a+ B-cells and (D) CD19+CD1c-CD1a- B-cells. Total CD19^+^ B-cells were gated on live CD45+ CDKPAN- cells (A). Relative frequencies of CD1c+CD1a-, CD1c+CD1a+ and CD1c-CD1a- populations were calculated vs total CD19+ B-cells (B, C and D). Data are presented as mean ± SD. Statistical significance of differences in the relative frequencies (%) were evaluated with Mann Withney U test when statistical no-parametric and with Unpaired T test when statistical parametric between HESNs and the two other groups. *p < 0.05 and **p < 0.01. HIV, human immunodeficiency virus; CSWs, commercial sex workers; HESN, HIV Highly-Exposed Seronegative. SD, standard deviation.

It has been shown that human MZ CD1c^+^ B-cells from blood, spleen and tonsils naturally bind to fully glycosylated gp120 through a process involving C-type lectins and/or polyreactive BCR [17], and in presence of BLyS/BAFF generate Ig, of which a fraction can recognize gp120. We thus analyzed whether genital CD1c^+^ B-cells could also bind to gp120. We found that the relative frequencies of MZ-like CD1c^+^ B-cells binding gp120 are higher than those that did not bind to gp120 (Fig 4B and 4C), whereas the majority of CD1c^-^ B-cells did not bind gp120 (Fig 4D), in all three groups of women. We found no significant differences in frequencies of CD1c and/or CD1a B-cell sub-populations binding gp120 between the different groups, albeit HESNs had higher frequencies of total B-cells binding gp120 than those found in the other groups (Fig 4A).

Overall, HESNs had lower relative percentages of innate MZ-like CD1c^+^ B-cells, whether they were CD1a^+^ or not, within their CVL cellular fraction when compared to both HIV-uninfected non-CSWs and HIV-infected CSWs. Most of the MZ-like CD1c^+^ B-cells bind to fully glycosylated gp120.

#### Concentrations of total, as well as gp120 and gp41 reactive immunoglobulin isotypes in the CVL supernatants of HIV-uninfected non-CSWs, HESNs and HIV-infected CSWs

**Fig 5:**
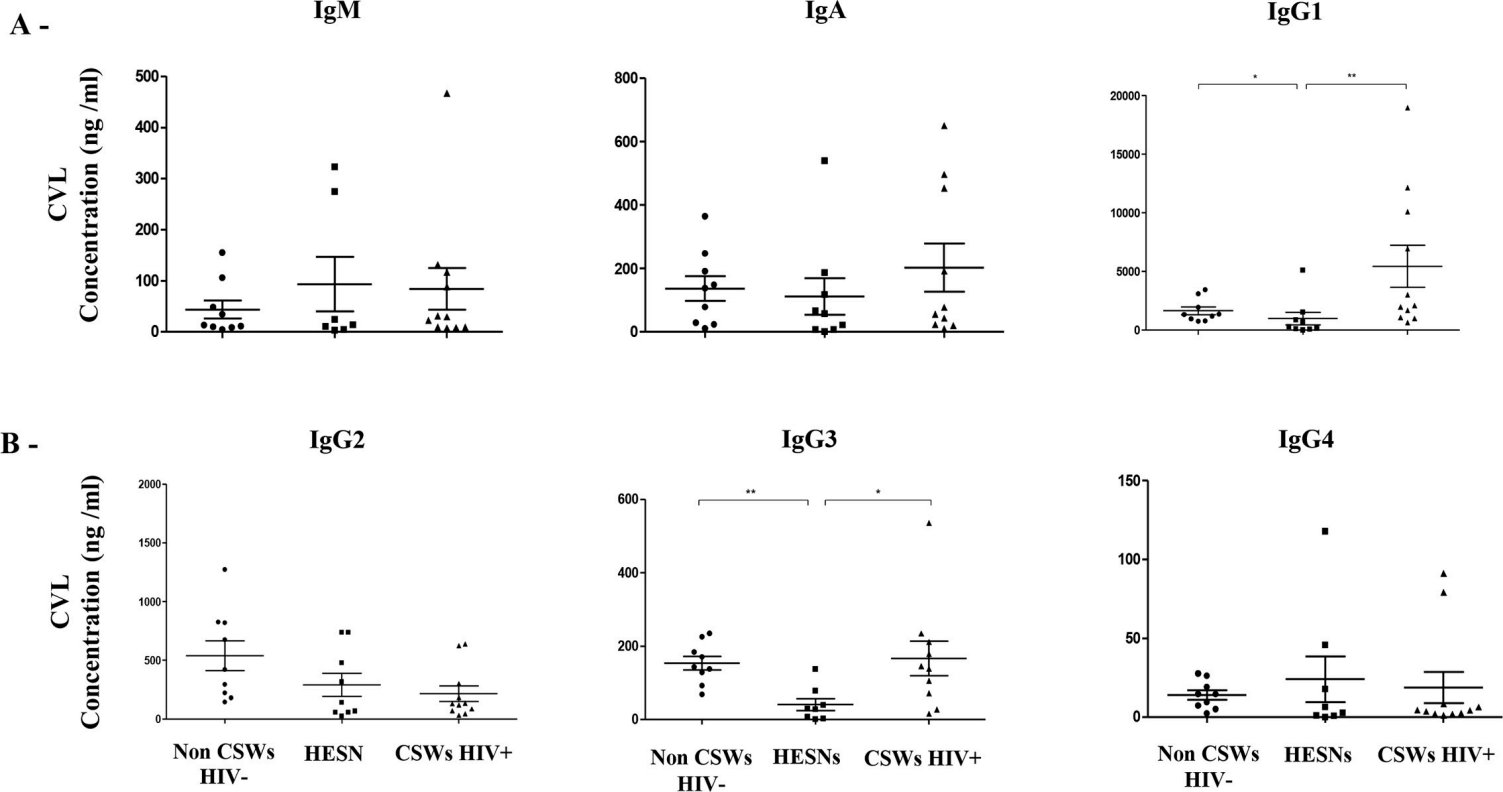
Total immunoglobulin (Ig) isotypes concentrations in cervicovaginal lavage (CVL) supernatants of HIV-1 uninfected non-CSW, HESN and HIV-1 infected CSW women. Immunoglobulin (A) M, A, G1 and (B) G2, G3, G4 isotypes concentrations (ng/ml) in CVLs were compared with Mann Whitney U test for pair-wise comparisons between HESNs and the two other groups. Data are presented as mean ± SD. Significance levels are shown as *(p < 0.05), **(p < 0.01), ***(p < 0.001). HIV, human immunodeficiency virus; CSWs, commercial sex workers; HESN, HIV Highly-Exposed Seronegative. SD, standard deviation.

**Fig 6:**
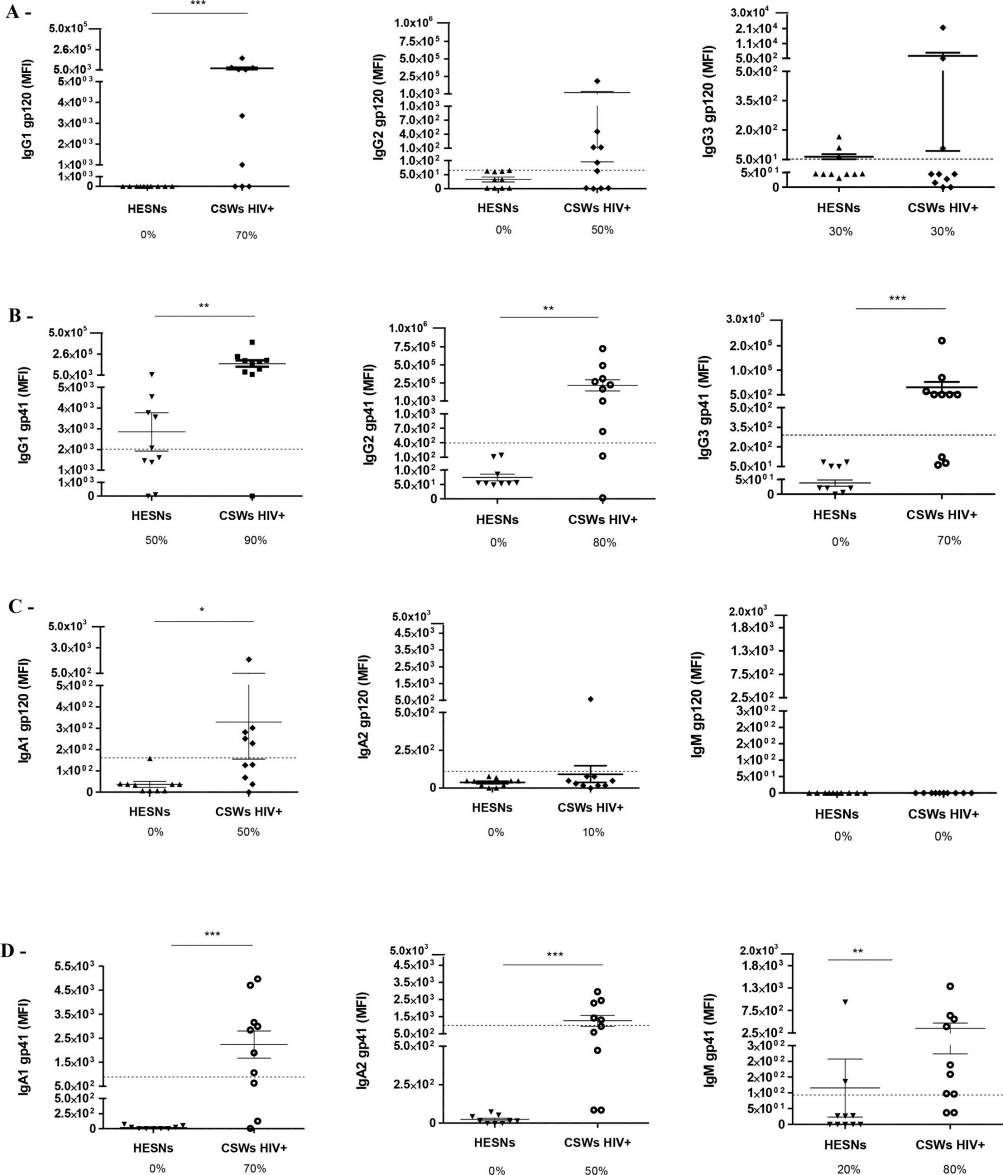
Levels of immunoglobulin (Ig) isotypes recognizing HIV-1 envelope proteins gp120 and gp41 in the cervicovaginal lavages (CVLs) of HESN and HIV-1 infected CSW women. Levels of immunoglobulin isotypes recognizing gp120 of HIV-1 (A) IgG1, IgG2 and IgG3, (C) IgA1, IgA2 and IgM, and immunoglobulin isotypes recognizing gp41 of HIV-1 (B) IgG1, IgG2 and IgG3, and (D) IgA1, IgA2 and IgM. Immunoglobulin isotypes levels were determined by Multiplex assay, data are presented as mean fluorescence intensity (MFI) as mean ± SD. Data were compared with Mann Whitney U test between HESNs and HIV-infected CSWs. Significance levels are shown as *(p < 0.05), **(p < 0.01), ***(p<0.001). The dotted line represents the cut–off value for positivity, which was set at MFI for 10 HIV-uninfected non-CSWs + 3 SD. Values below the dotted line were considered as negative. Detection rates are expressed as %. HIV, human immunodeficiency virus; CSW, commercial sex worker; HESN, HIV Highly-Exposed Seronegative; gp, glycoproteins. SD, standard deviation.

We have previously shown that HIV-infected CSWs present increased frequencies of IgG^+^ plasmablasts in their blood and hypergammaglobulinemia in their serum when compared to HESNs [10]. The evaluation of total immunoglobulin isotypes in CVL supernatants demonstrated that HESNs had significantly lower concentrations of total IgG1 and IgG3 when compared to both HIV-uninfected non-CSWs and HIV-infected CSWs (Fig 5A and 5B). There were no significant differences in total IgM and IgA levels between the groups. IgG1 and IgA1 reactivity to both gp120 and gp41 (Fig 6A and 6C left panels and Fig 6B and 6D left panels), as well as IgG2, IgG3, IgA2 and IgM reactivity to gp41 (Fig 6B and 6D middle and right panels) were observed in CVL supernatants of the majority of HIV-infected CSWs. No Ig reactivity to gp120 and gp41 were detected in the CVL supernatants of HESNs (Fig 6), except for low IgG1 (Fig 6B left panel) and IgM (Fig 6D right panel) reactivity to gp41 in some individuals. Overall, we found that gp120 reactivity in CVLs of HIV-infected CSWs mostly involved IgG1 and IgA1 isotypes, whereas gp41 reactivity involved all isotypes. We found gp41 reactivity in a proportion of HESNs, which was mainly of the IgG1 isotype.

## Discussion

We [2, 3, 9] and others [8, 18, 19] have shown that HESN female CSWs have lower genital inflammation when compared to both HIV-infected CSWs and HIV-uninfected non-CSWs. We hypothesized that maintenance of low-inflammatory conditions in the female genital tract of HESN individuals may help to prevent excessive immune activation and lower HIV target availability, likely maintaining the integrity of the mucosal barrier to protect from HIV infection [4, 20]. In agreement with this, HESNs had lower blood [10] and genital levels of soluble BLyS/BAFF and lower frequencies of BLyS/BAFF expressing cells in their genital mucosa. In contrast, the genital cells of HESNs expressed relatively higher levels of BLyS/BAFF than the cells of the other groups of women. These observations are suggesting that upregulation of BLyS/BAFF expression seems required, but regulated as to prevent deleterious effects. Recent studies have shown that plasmacytoid DCs exposed to HIV in vitro upregulate BLyS/BAFF cell surface expression without releasing the molecule [21]. This raises the possibility that the low levels of BLyS/BAFF measured in blood [10] and CVL supernatants of HESNs may be linked to the signals leading to BLyS/BAFF release. As to whether these are related to advantageous genetic polymorphisms remain to be established. We have recently analyzed BAFF promoter -871, -2841 and -2701 mutations associated with elevated BLyS/BAFF plasma levels and susceptibility to auto-immune diseases such as Systemic Lupus Erythematosus and hepatitis C associated cryoglobulinemia [22–24] in our Benin cohort and found no association between BAFF promoter mutations and either blood and CVLs BLyS/BAFF levels nor HIV infection (S4 Fig).

The relatively high levels of BLyS/BAFF observed in the blood [10] and CVL supernatants of HIV-infected CSWs are consistent with our previous reports for HIV-infected rapid and classic progressors [16], and likely due to direct and indirect factors associated with HIV infection [20]. Plasma [10] and CVL levels, but not cell surface expression, of BLyS/BAFF measured in HIV-uninfected non-CSWs were similar to those observed in HIV-infected CSWs. This may be due to inflammatory/infectious conditions other than HIV in HIV-uninfected non-CSWs that can stimulate soluble release of BLyS/BAFF [10].

Consistent with lower levels of soluble BLyS/BAFF, HESNs had reduced frequencies of innate MZ-like CD1c^+^ B-cells in their genital tract when compared to HIV-infected CSWs and HIV-uninfected non-CSWs. Growing importance is given to innate MZ B-cells in health and disease [11], as they constitute early first-line defense against invading pathogens and participate in the development of adaptive antibody (Ab) responses by trafficking to follicular B-cell areas of lymphoid structures and promoting germinal center reactions [25]. MZ B-cells are capable of isotype switching and can present a somatically mutated pre-diversified low affinity polyreactive BCR repertoire [11], which comprises usage of the IGHV1-2 gene [26], shown to take part in HIV-ENV reactive broadly neutralizing Abs (bNAbs) such as VRC01 [27]. Interestingly, repeated treatment of mice with BLyS/BAFF increased their MZ compartment, and generated an increased response to ENV immunization and bNAbs [28]. In agreement with the observations made by Cerutti and colleagues [17], the innate/MZ-like CD1c^+^ B-cell populations we identified in the genital tract of Beninese women also bind to fully glycosylated gp120, and to a greater frequency than CD1c^-^ B-cells. Although this suggests that these cells have the capacity to transfer HIV to target cells, it is unlikely that they get infected by the virus since it has not yet been convincingly shown to infect or replicate in B-cells in vivo [29]. Interestingly, although they have lower frequencies of CD1c^+^ MZ-like B-cells, HESNs have higher relative frequencies of total B-cells binding gp120 when compared to the other groups. The fact that there were no significant differences in frequencies of CD1c^+^ B-cell sub-populations binding gp120 between the different groups suggests that in HESNs, genital B-cells other than CD1c^+^CD1a^-^ and CD1c^+^CD1a^+^ subsets have a greater capacity to bind gp120 that those in HIV-infected CSWs and HIV-uninfected non-CSWs. It is possible that the relative binding capacity was lower in the HIV-infected CSWs because gp120 receptors were saturated in these individuals. In contrast to that observed by He et al [17], pre-incubation of total B-cells with mannose did not significantly diminish gp120 binding (S2B Fig), suggesting receptors of various types might be involved. Identifying these receptors and B-cell sub-population(s) binding gp120 that are increased in HESNs will require further experimentation. Also, the exact nature of the innate MZ-like CD1c^+^ B-cells we identify in the genital tract has yet to be confirmed, and as to whether they have a direct link with those we previously observed in blood [10] remains to be established.

In humans, MZ B-cells recirculate and have been found in front-line areas such as the sub epithelial lamina propria of mucosal associated lymphoid tissues (MALT) [11]. To our knowledge, we show for the first time that MZ-like B-cells can be found in the female genital tract, which is part of the MALT and is populated by a commensal microflora [4, 30]. It is thus conceivable that the innate MZ-like CD1c^+^ B-cells observed in the genital tract of Beninese women participate in local immune responses to control microflora and pathogens. Moreover, it has been shown that the gut lamina propria can be a T-independent inductive site in humans [31] and likely similar mechanisms operate at the genital lamina propria. The recent characterization of elevated BLyS/BAFF levels and transient Gp41-specific IgA in mucosal genital fluids from patients within the first weeks after HIV transmission, suggest that these Abs might have originated from first-line B-cell populations [32]. The fact that HESNs who undergo sex-break eventually seroconvert [33], suggests natural immunity involves populations of which pool maintenance in the genital mucosal niche requires frequent antigen exposure, and this is consistent with first-line responses. As to whether first-line responses are actually polyreactive with shared HIV-specificity and/or involve shared antigenicity with the local microbiota remains to be determined.

Depending on the level of inflammation, CD1c^+^ B-cells may contribute to natural immunity against HIV or conversely promote disease progression. Indeed, as shown previously [10, 16], elevated BLyS/BAFF levels increase expansion, activation and dysregulation of innate B-cell populations such as precursor-like MZ B-cells, likely contributing to the over-representation of low affinity, polyreactive and auto-reactive Abs [34] at the expense of high affinity polyfunctional eradicating anti-HIV Ab responses. As such, we found hyperglobulinemia in the blood [10] and CVL supernatants of HIV-infected CSWs. Although we found no significant difference in the frequencies of total or IgG^+^ plasmablasts/plasma cells in the genital tract of Beninese women, relative percentages of total CD138^+^CD1c^+^CD1a^+^ cells bearing IgG were significantly higher in HIV-infected CSWs when compared to HESNs. It has yet to be determined whether these cells are substantially involved in the relatively high IgG1 and IgG3 levels measured in the CVLs of HIV-infected CSWs.

Most genital immunoglobulins (Ig) are found in the mucus [35], unfortunately the latter was removed prior to CVL sample collection in our study. Nevertheless, IgG1 and IgA1 reactivity to both gp120 and gp41, as well as IgG2, IgG3, IgA2 and IgM reactivity to gp41 were observed in CVL supernatants of the majority of HIV-infected CSWs. However, despite the elevated frequencies of B-cells binding to gp120, we found no Ig reactivity to gp120 in the CVL supernatants of HESNs. It is possible that lower levels of Ig are present in the samples of HESNs but mucus removal has precluded their detection. Accordingly, we have previously detected anti-HIV-1-Env-specific IgG, neutralizing or ADCC activities in blood and CVL samples from HIV-infected CSWs but not in those from HESNs [36]. Interestingly, we could detect IgG1 reactivity to gp41 in some HESNs, which could be derived from a microbiota reactive, possibly first-line B-cell pool [37], as most gp41 reactive Abs cross-react with microbiota [38]. Whether the gp41 binding IgG1 Abs detected in the CVL of HESNS can confer some level of protection remains to be established. There is increasing evidence for non-neutralizing functions of antibodies in decreasing the viral load, and in conferring some level of protection [39]. In this view, anti-gp41 IgG antibodies are found in the plasma of HIV-infected individuals shortly after transmission, and form antibody-virion complexes, which although ineffective at controlling disease progression [40], have been associated with infectivity decay [41]. We could not detect substantial IgA1 and IgA2 reactivity to gp120 or gp41 in the CVL supernatants of HESNs. To date, studies have reported contradictory results regarding the presence of anti-HIV specific IgA responses in the genital tract of HESNs [42–47]. The discrepancies between studies may be due to relatively small sample size of these studies and/or the different techniques used to detect ENV-reactive Abs.

Because of the cross-sectional design, the present study cannot address whether the lower levels of BLyS/BAFF and CD1c^+^ B-cells, as well as gp41 reactive IgG1 found in the genital tract of HESNs have a protective role against HIV infection. Comparison between HESN and women involved in sex work but not yet HESN should also be done to control the effects of sex work itself on genital immunology. Longitudinal studies and further phenotypic and functional characterizations are required to confirm a protective role, and the exact nature of genital CD1c^+^ B-cells and their responses.

To wrap-up, understanding the dynamics of BLyS/BAFF and its role in homeostasis of immune responsiveness appears pivotal to the design of vaccine strategies soliciting first-line B-cell responses to help protect from HIV infection. Based on our observations, the capacity to contain BLyS/BAFF expression levels seems concomitant with natural immunity against HIV, whereas excessive BLyS/BAFF may promote immune dysregulation, risk of infection and disease progression. The fact that human genital innate MZ-like B-cells naturally bind to fully glycosylated gp120 renders these cells of particular interest because MZ B-cells can acquire Ig somatic mutations and could be harnessed to increase HIV-ENV affinity.

## Supporting information

S1 Fig Flow-Cytometry gating strategy for analysis of cells from cervico-vaginal lavages (CVLs). (TIF)

S2 Fig (A) RNA-Seq analyses of CD1a expression by exvivo human blood marginal zone (MZ) and precursor-like MZ B-cells. Data are presented as the mean value of samples from 3 healthy donors ± SD. (B) Cells were pre-incubated (dark circles) or not (clear circles) with mannose (5 ug) for 40 minutes on ice, followed by incubation with fully glycosylated biotinylated gp120 IIIB at 5 ug/ml for 40 minutes on ice prior to adding the staining cocktail and streptavidin-PE. Data are presented as percentage (%) of gp120 binding for total B-cells (top panel), CD1c+CD1a- B-cells (middle panel) and CD1c+CD1a+ B-cells (lower panel). Statistical significance of differences were evaluated with Mann Withney U test when statistical no-parametric and with Unpaired T test when statistical parametric between HESNs and the two other groups. HIV, human immunodeficiency virus; CSWs, commercial sex workers; HESN, HIV Highly-Exposed Seronegative.
(TIF)

S3 Fig Flow-Cytometry gating strategy for analysis of B-cells from cervico-vaginal lavages (CVLs). (TIF)

S4 Fig BLyS/BAFF expression levels in cervico-vaginal lavages (CVLs) do not correlate with BLyS/BAFF promotor polymorphisms in regions -871, -2701, -2841, amongst the three study groups. (WT: Wild Type; MT: Mutant)
(TIF)
