# Supplementary figures and images for: Natural Immunity to HIV is associated with Low BLyS/BAFF levels and low frequencies of innate marginal zone like CD1c+ B-cells in the genital tract
Source: PLoS Pathog. 2019 Jun 7;15(6):e1007840. doi: 10.1371/journal.ppat.1007840 (PMC6583986; doi:10.1371/journal.ppat.1007840)

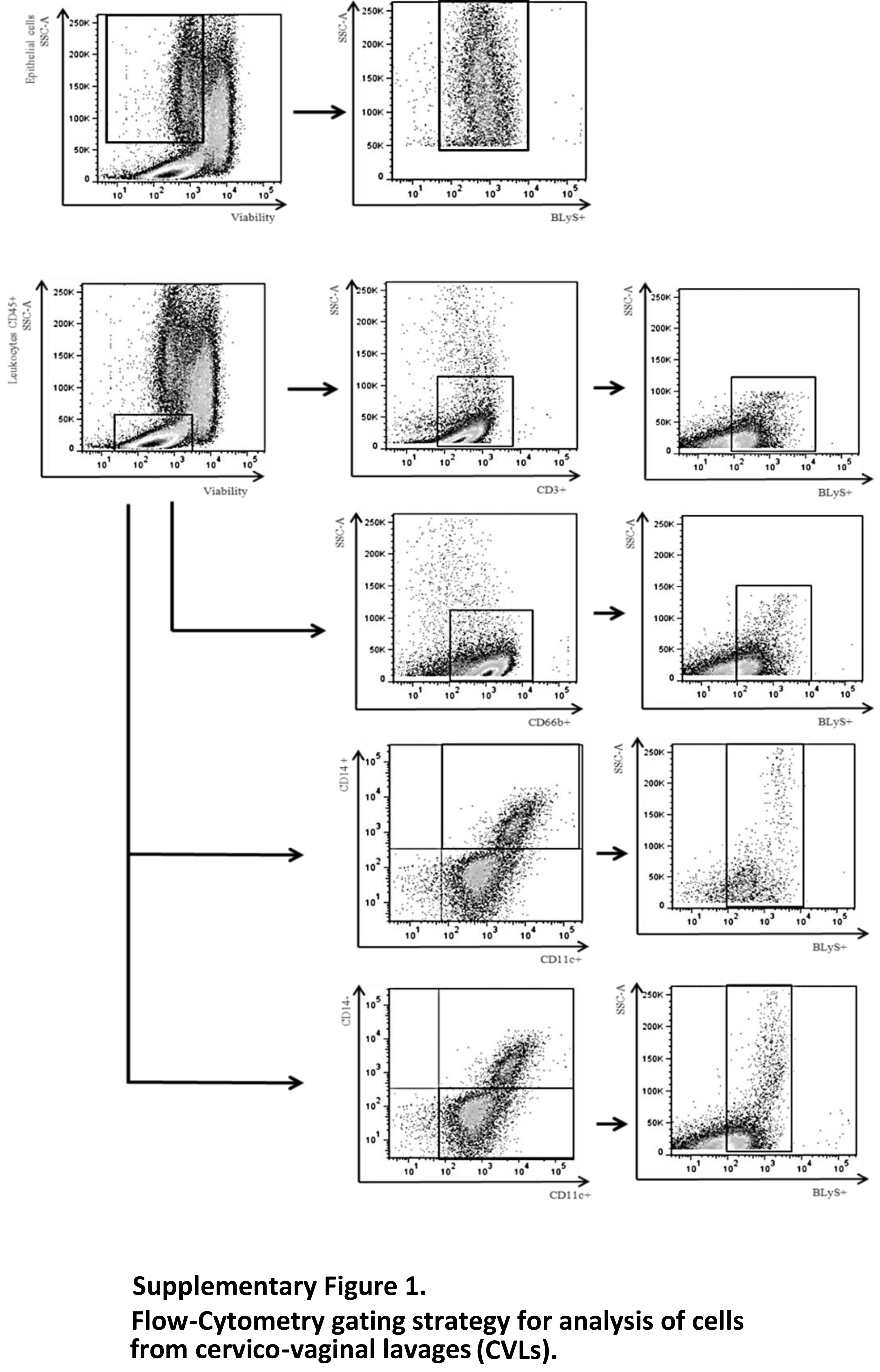

Supplement: S1 Fig — (TIF) [file ppat.1007840.s001.tif]

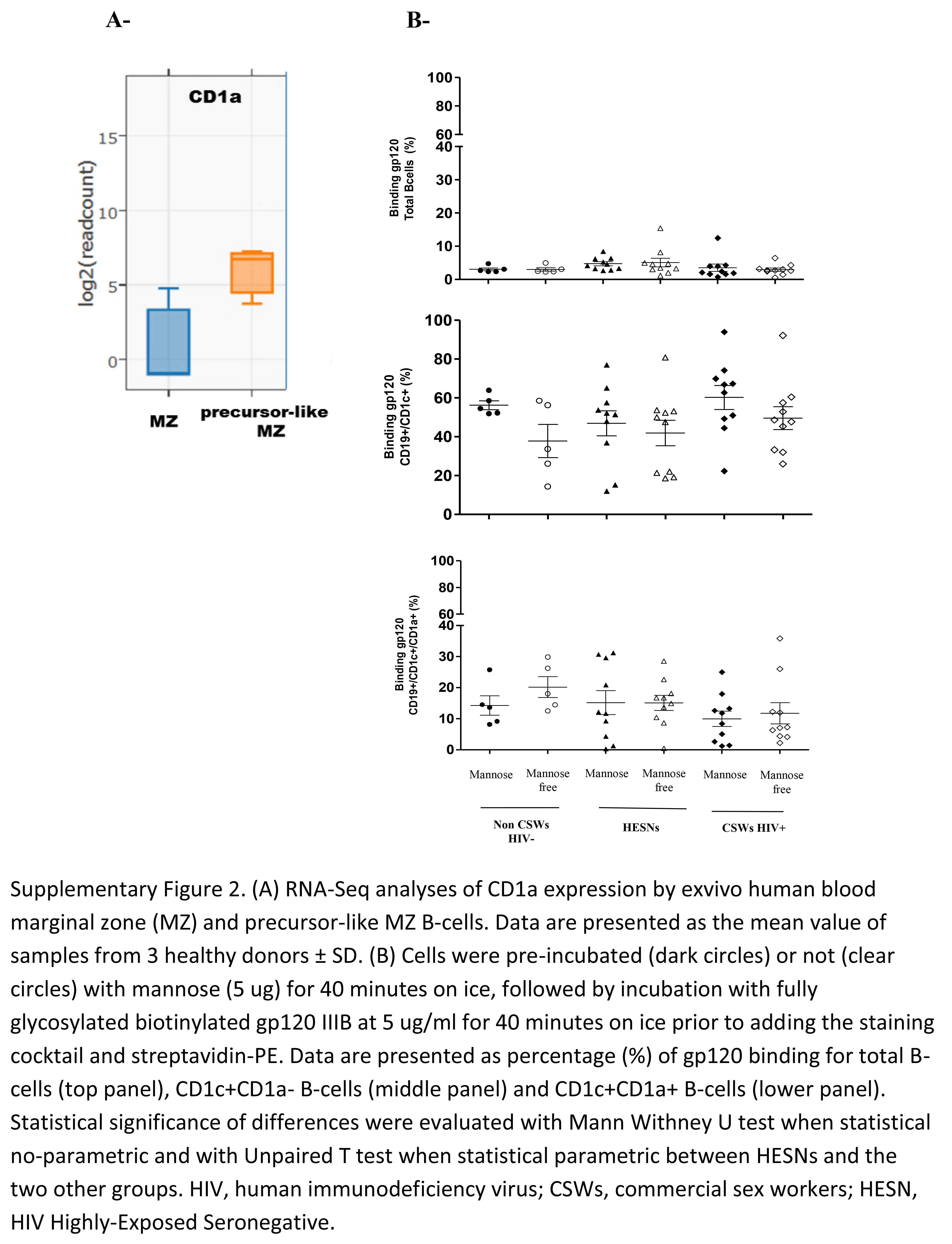

Supplement: S2 Fig — (A) RNA-Seq analyses of CD1a expression by exvivo human blood marginal zone (MZ) and precursor-like MZ B-cells. Data are presented as the mean value of samples from 3 healthy donors ± SD. (B) Cells were pre-incubated (dark circles) or not (clear circles) with mannose (5 ug) for 40 minutes on ice, followed by incubation with fully glycosylated biotinylated gp120 IIIB at 5 ug/ml for 40 minutes on ice prior to adding the staining cocktail and streptavidin-PE. Data are presented as percentage (%) of gp120 binding for total B-cells (top panel), CD1c+CD1a- B-cells (middle panel) and CD1c+CD1a+ B-cells (lower panel). Statistical significance of differences were evaluated with Mann Withney U test when statistical no-parametric and with Unpaired T test when statistical parametric between HESNs and the two other groups. HIV, human immunodeficiency virus; CSWs, commercial sex workers; HESN, HIV Highly-Exposed Seronegative. (TIF) [file ppat.1007840.s002.tif]

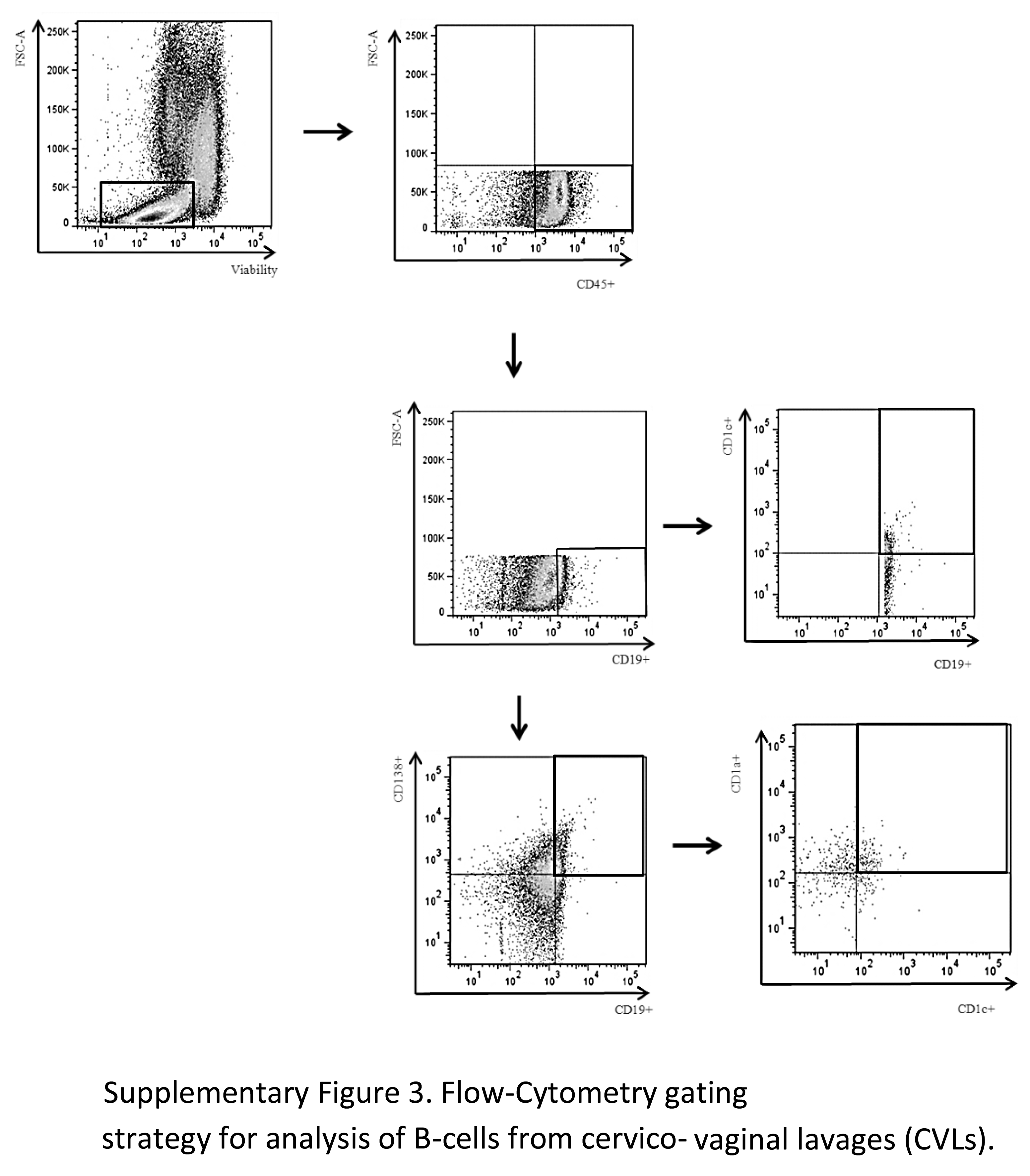

Supplement: S3 Fig — (TIF) [file ppat.1007840.s003.tif]

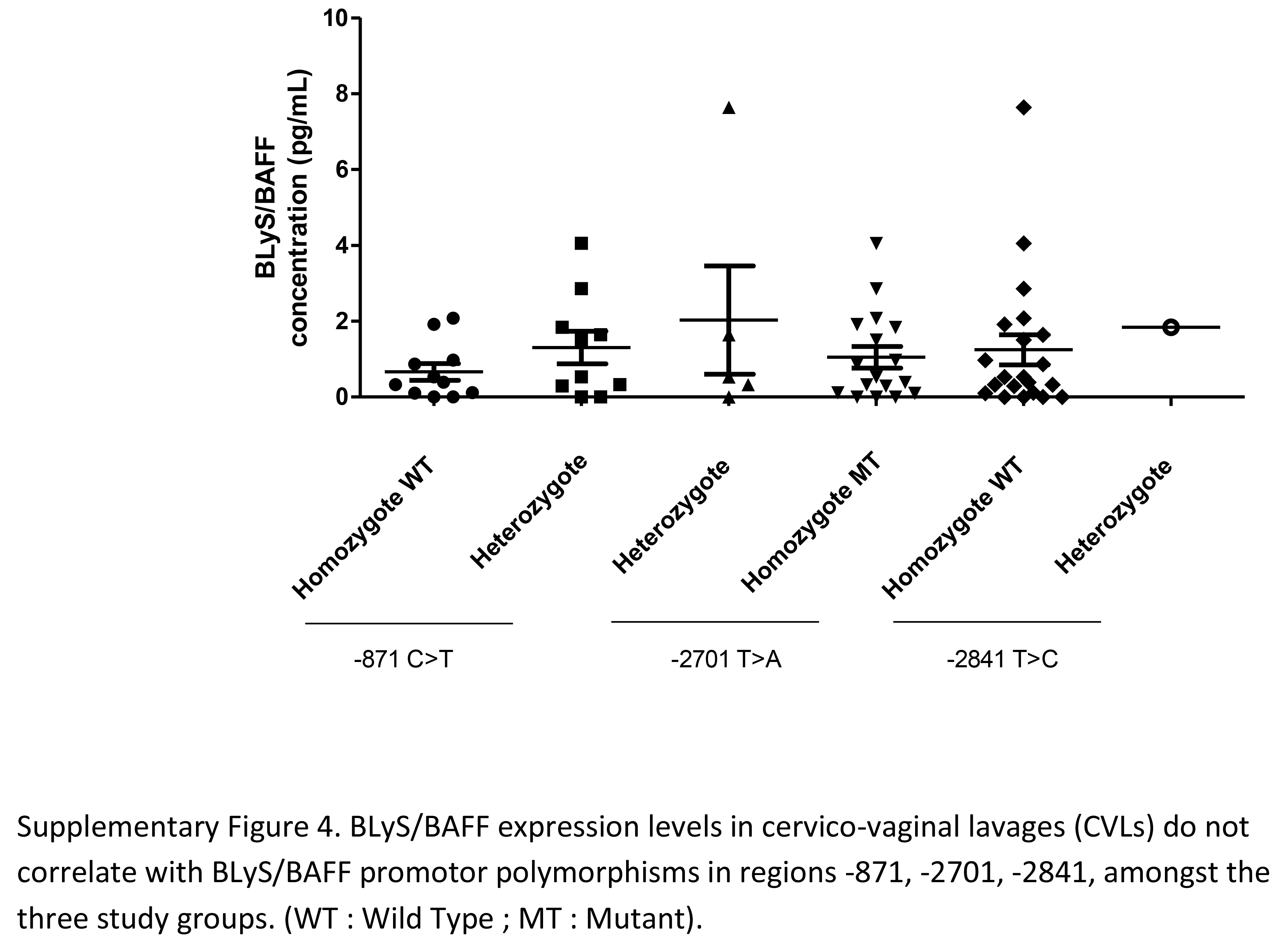

Supplement: S4 Fig — (WT: Wild Type; MT: Mutant) (TIF) [file ppat.1007840.s004.tif]
